# Space-time evolution of urban flood resilience and its scenario simulation research: A case study of Zhejiang Province, China

**DOI:** 10.1016/j.heliyon.2025.e42698

**Published:** 2025-02-13

**Authors:** Feifeng Cao, Hao Xu, Guixia Huang, Conglin Zhang

**Affiliations:** a College of Civil Engineering, Zhejiang University of Technology, Hangzhou, 310023, Zhejiang, China; b Zhejiang Yongbang Emergency Technology Co., Ltd, Hangzhou, 310030, Zhejiang, China; c Institutes of Science and Development, Chinese Academy of Sciences, Beijing, 100190, China; d School of Public Policy and Management, University of Chinese Academy of Sciences, Beijing, 100190, China

**Keywords:** Urban flood resilience, Space-time variation, Evaluation, Indicator, Scenario simulation

## Abstract

Recent years have seen a surge in global flood disasters, underlining the imperative for urban areas to enhance flood resilience. To quantitatively evaluate the capacity of urban areas to manage flood disasters and model the evolving trend of urban flood resilience, a system was established for evaluating urban flood resilience. This system utilized both the global entropy method and sensitivity analysis for evaluation and simulation purposes. In light of the intricate and multifaceted factors influencing urban flood resilience, and by incorporating *the Guide for Safety Resilient City Evaluation* along with key indicators identified in Chinese and international research, we developed an evaluation indicator system for urban resilience in dealing with flood disasters. Moreover, the global entropy method was utilized to derive the urban flood resilience indices. The study developed four scenarios to analyze varying resilience trajectories. Focusing on Zhejiang Province, a region with frequent and representative flood occurrences, the indicator system, which is constructed by the aforementioned method, was applied to evaluate its urban flood resilience from 2007 to 2021. The resilience evolution under these scenarios was also explored. The results reveal an overarching positive trend in Zhejiang Province's resilience across natural, economy, and infrastructure dimensions, with consistently high social resilience. In the absence of external disruptions, all cities within Zhejiang Province are projected to continue enhancing their flood resilience, exceeding an annual growth rate of 1.5 %. Building on these insights, the study identified weaknesses of various cities within Zhejiang Province under each scenario, offering targeted recommendations for resilience enhancement.

## Highlights

• The study of the space-time evolution of urban flood resilience has been enriched.
• The indicator system considers local feature, standard, and key common indicators.
• The varying resilience trajectories are analyzed from different scenarios.

## 1 Introduction

The interplay between population growth, the rise in extreme weather events, and rapid urbanization has resulted in frequent urban flood disasters in numerous cities worldwide [1,2]These disasters profoundly impact urban living environments, economic growth, social stability, and infrastructure integrity. For example, the 2021 floods in China affected approximately 59.01 million people, led to 590 casualties or missing persons, and resulted in direct economic losses of 245.892 billion yuan [3] In response to these threats, the concept of urban resilience has gained prominence, focusing on enhancing urban systems' capability to resist and recover from flood-related challenges.

Urban resilience, though lacking a universally accepted definition, is broadly recognized as a city's capacity to withstand pressures, maintain essential structures and functions, adapt to challenges, and recover from disruptions[[4], [5], [6]]. Thus, urban flood resilience encompasses a city's ability, across natural, economy, society, and infrastructure dimensions, to manage and rebound from flood disasters. To construct resilient cities capable of coping with flood disasters, it is crucial to quantitatively evaluate the level of urban flood resilience and analyze its spatial and temporal evolution patterns. Quantitatively evaluating urban flood resilience helps to clarify the current stage of flood resilience in cities. Analyzing the spatial and temporal evolution patterns of urban flood resilience helps in understanding the resilience characteristics over time and across different regions. Moreover, simulating urban flood resilience scenarios enables the examination of resilience under various conditions, providing insights into dynamic changes. These initiatives are vital for protecting lives and property and promoting sustainable urban development.

## 2 Literature review urban flood resilience research currently centers on three primary aspects

Regional studies: Urban scale: Investigations cover various cities including Shanghai [7], Nanjing [8], Guangzhou [2,9], and Shenzhen, China [[10], [11], [12]]; Arnhem, Netherlands [13]; Surat, India [14]; Norfolk, USA [15]; Isfahan, Iran [16]. Provincial/state scale: Examples include South Sumatra Province, Indonesia [17] and Louisiana State, USA [18]. National scale: Studies in countries like China [19,20], South Africa [21], and Sri Lanka [22]are notable. Predominantly, research is more abundant at the urban scale compared to provincial/state or national scales.

Research methods: Resilience evaluation: The predominant method is the indicator method [7,21,23,24], which selects and constructs indicators for a more realistic evaluation. Other methods enhancing this approach include the wind-driven optimization algorithm (WDO) [25], natural breakpoint classification [2,26], NDVI index [27], and computer model analysis [11,14]. These methods significantly enhance the approach to evaluating resilience and enhance its accuracy.

However, the progression and transformation of urban resilience represent an intricate and ever-changing process. In order to better study this process, some scholars choose to integrate scenario construction into resilience evaluation, simulating a certain development scenario of the city through scenario construction, obtaining its development situation, and providing a more reasonable reference for the development of the city. Some scholars have employed scenario simulation techniques to investigate regional ecological resilience, thereby offering a more precise and comprehensive insight into the future dynamics of local ecological resilience [28,29]. There are also scholars that concentrate on the risks encountered in urban development. By utilizing scenario simulations, these scholars correlate urban water usage with energy demand, and integrate the dynamics of water and energy risks within cities. This approach yields distinctive insights into the pursuit of sustainable urban development [30]. Scenario construction: This involves creating simulations to project urban development trends. The primary method used is sensitivity analysis[25,[30], [31], [32]], optimizing key indicators to construct research scenarios. Other methods include ontology-based methods [33], group decision simulations [34], and case analysis [35]. These methods can all help construct the intended research scenarios and offer valuable insights for the investigation of urban resilience development. Nevertheless, there are still certain limitations in their current utilization within the field of urban flood resilience research.

Research conclusions: Studies have evaluated spatial differences in urban flood resilience within and between cities [9,13,20] and provided suggestions for resilient city development, including policy, land use, urban planning, and resident rights protection. Research has also focused on the applicability of evaluation indicators or methods [22,36,37], testing new systems or approaches while evaluating resilience levels. Significant factors influencing urban flood resilience have been identified[[38], [39], [40]], such as economic aspects, rainfall patterns, and land types, aiding in the efficient management of urban flood resilience.

In summary, there have been significant advancements in urban flood resilience research. Nonetheless, several areas still require further enhancement: (1) Inadequate exploration of the space-time evolution of flood resilience in hilly regions with long coastlines, abundant rainfall, dense river networks, frequent typhoons, high population density, and a subtropical monsoon climate. Such regions are found in East Asia, South Asia, North America, and other areas. (2) Limited research on urban flood resilience using scenario simulation approaches. (3) The necessity to enhance the applicability and scientific rigor of the current indicator system for evaluating urban flood resilience.

## 3 Methods

### 3.1 Determination of the evaluation indicator system

#### 3.1.1 Characterization of flood resilience in the study area

**Fig. 1:**
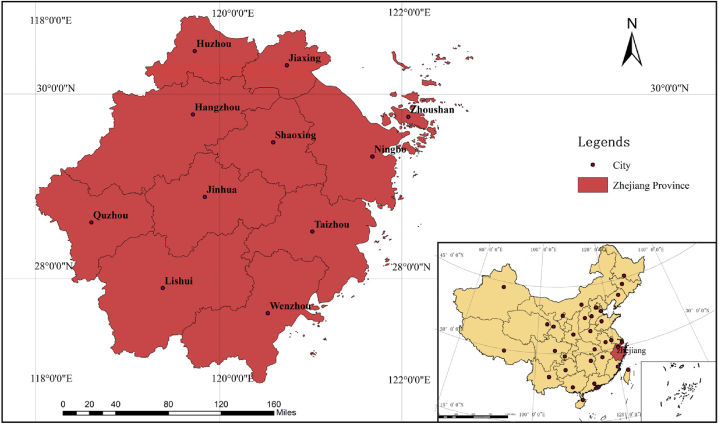
Geographic location of Zhejiang Province.

The high rainfall concentration, extensive coastline, and frequent flood disasters in Zhejiang Province render it a representative case for studying resilience along the southeastern coast of China [41]. Analyzing the levels of flood resilience and their evolving trends in various cities within Zhejiang Province holds significant importance in evaluating flood resilience and building resilient cities in similar regions worldwide. In this paper, we aim to define urban flood resilience and intend to evaluate the existing flood resilience attributes of Zhejiang Province by considering four dimensions: natural, economy, society, and infrastructure, taking into account the province's actual conditions (Fig. 1).

Regarding the natural aspects, Zhejiang Province is characterized by predominantly hilly terrain, featuring significant variations in topography across different regions. Zhejiang Province falls under a monsoon climate, experiencing approximately two months of rainy season each year with concentrated rainfall. This makes the region highly susceptible to flooding. Additionally, areas such as Zhoushan, Wenzhou, and Taizhou are also prone to typhoons, which further intensify the flooding risk. When evaluating flood resilience, taking into account regional factors like topography and rainfall patterns can effectively illustrate the variations in flood resilience across different areas within the province. Approximately 70 % of the land in Zhejiang Province is classified as forest land, with Lishui, Hangzhou, and Wenzhou alone accounting for over half of the province's total forest land area. Compared to built-up areas, forest land plays a crucial role in reducing flood peaks and enhancing regional flood resilience. However, in recent years, rapid urbanization has led to an increase in construction land across various regions, and other types of land use have also undergone continuous changes. Therefore, it becomes essential to consider the impact of land use type changes on regional flood resilience, given their potential to influence the overall resilience of the area.

In the economic aspect, Zhejiang Province has experienced an average economic growth rate of 6.7 % over the past five years, and the level of economic development significantly affects local flood defense investments. Moreover, the proportion of primary industry varies across different parts of Zhejiang Province and is subject to ongoing adjustments. The primary industry plays a substantial role in influencing flood resilience capacity [25]. Therefore, it is crucial to take into account the impact of changes in industrial structures on regional flood resilience, as these changes can have a notable effect on the region's ability to withstand floods.

In terms of the social dimension, as of the end of 2021, Zhejiang Province had a resident population of 65.77 million, with approximately half of this population concentrated in Hangzhou, Ningbo, and Wenzhou. The province is characterized by high population density, with significant variations in population density among its cities. Population density is a crucial indicator in resilience research because areas with high population density face greater pressure during evacuation and rescue operations. Including population density in the study can provide a more detailed understanding of the role played by the population in urban flood resilience. Additionally, in 2021, Zhejiang Province had a significant portion of its population composed of minors and individuals over the age of 60, accounting for about 41 % of the total population. The male-female sex ratio was 109 males for every 100 females. Vulnerable groups, such as the elderly, minors, and women, are particularly at risk during flooding events. Therefore, it is essential to focus on these vulnerable groups and evaluate the impact of flooding on their well-being and safety.

In the realm of infrastructure, Zhejiang Province places significant emphasis on the development of water conservancy infrastructure. In 2021, the province invested nearly 64 billion yuan in water conservancy infrastructure, recognizing its pivotal role in flood prevention and control, ultimately enhancing the city's resilience against flooding. Furthermore, in 2021, Zhejiang Province boasted a total urban water supply pipeline length of 97,310 km and a drainage pipeline length of 60,698 km. These drainage networks play a critical role in mitigating floods within cities [42]. Additionally, in 2021, the urban green coverage area in Zhejiang Province reached 2054.24 km^2^. Urban green spaces serve as effective interceptors of rainfall, reducing flood peaks, and consequently, enhancing a city's ability to withstand flooding events. Thus, it is essential to consider the impacts of hydrometeorological facilities, urban pipe networks, and green spaces when evaluating urban flood resilience.

#### 3.1.2 Key common evaluation indicators identified in relevant studies

In urban flood resilience research, numerous evaluation indicators have been proposed, with some recurring frequently and significantly influencing the evaluation results. By analyzing existing studies, we can identify key common indicators, ensuring a scientific and practical approach to indicator selection. These indicators are categorized into four aspects: natural, economy, society, and infrastructure. They are ranked based on their frequency of appearance in the literature, with the findings presented in Table 1.

**Table 1:** Key common evaluation indicators proposed in relevant studies.

Primary indicator	Secondary indicator	Frequency	References
**Nature (N)**	Land use (N1)	11	[[12], [13], [14],^17^,[19], [20], [21], [22],^25^,^27^,^37^]
	Topography (N2)	7	[2,14,16,22,27,40,43]
	Rainfall (N3)	6	[2,13,14,22,25,26]
**Economy (E)**	Income of residents (E1)	12	[2,7,8,13,14,17,21,25,26,38,44,45]
	Insurance (E2)	6	[18,26,40,[45], [46], [47]]
	Financial expenditure (E3)	6	[7,13,20,27,40,45]
	Economic diversity (E4)	5	[7,8,25,40,47]
**Society (S)**	Education (S1)	12	[10,13,17,18,20,21,26,27,40,43,46,47]
	Population (S2)	10	[7,14,[19], [20], [21],[25], [26], [27],^44^,^46^]
	Age structure (S3)	9	[7,10,13,18,21,26,40,44,46]
	Medical level (S4)	8	[2,7,10,20,26,27,40,45]
	Gender (S5)	6	[7,10,17,18,44,47]
	Flooding knowledge (S6)	5	[23,40,[45], [46], [47]]
**Infrastructure (I)**	Transportation (I1)	10	[2,8,10,21,25,26,35,40,45,46]
	Water infrastructure (I2)	7	[7,8,20,23,25,26,40]
	Green area (I3)	6	[2,7,8,23,26,46]
	Pipe networks (I4)	6	[8,25,35,37,40,45]
	Communication infrastructure (I5)	5	[18,20,35,40,45]

#### 3.1.3 Key indicators in existing national standards

The evaluation of urban flood resilience can be informed by relevant standards, such as *the Guide for Safety Resilient City Evaluation* published by the China National Committee for Public Safety Standardization Technology. This national standard serves as a highly valuable reference for evaluating urban flood resilience. In this study, we chose indicators from the guidelines based on their practicality, correlation, differentiation, and data availability. The ultimate selection of indicators involved opting for the lowest rating of the four dimensions serves as the final evaluation of the indicator. The results of this indicator selection process are detailed in Table 2.

**Table 2:** Results of the screening of indicators for *the Guide for Safety Resilient City Evaluation*.

Primary indicator	Secondary indicator	Practicality of indicators	Correlation of indicators	Differences in indicators	Availability of data	Availability of indicators
**Society (s)**	Proportion of employed population with tertiary education (s1)	☆	△	☆	☆	**△**
	Age (s2)	☆	☆	☆	☆	**☆**
	Basic medical insurance coverage (s3)	☆	△	☆	△	**△**
	Proportion of population with disabilities (s4)	☆	☆	☆	☆	**☆**
**Infrastructure (i)**	Land development intensity (i1)	☆	△	☆	☆	**△**
	Road area per capita (i2)	☆	△	☆	☆	**△**
	Meteorology, flood monitoring (i3)	☆	☆	☆	△	**△**
	Green coverage (i4)	☆	☆	☆	☆	**☆**
	Mobile phone penetration rate (i5)	☆	△	☆	☆	**△**

Practicality refers to the local existence of an indicator, correlation to its relevance to flood resilience evaluation, and differentiation to its variability across regions or over time. Ratings are assigned as follows: “☆” for full compliance, “△” for general compliance, and “○” for non-compliance. Indicators rated “☆” and “△” are considered useable, while those with a “○” rating are deemed non-useable.

#### 3.1.4 Indicator system for evaluating urban flood resilience in Zhejiang province

Taking into account the analysis of essential indicators in prior research and the key indicators in existing national standards, we have compiled a comprehensive evaluation indicator system for urban flood resilience in Zhejiang Province, aligning it with the fundamental characteristics of flood resilience in the region. This system is outlined in Table 3.

**Table 3:** Indicator system for evaluating urban flood resilience in Zhejiang Province.

Primary indicator	Secondary indicator	Explanation of indicators	Indicator positive/negative	Reflecting local characteristics	Reflecting the key common evaluation indicators in relevant studies	Reflecting the key indicators in existing national standards
**Nature (A)**	Land use (A1)	Percentage of built-up areas	–	Nature	N1	i1
	Topography (A2)	Percentage of terrain with slopes of less than 5°	+	Nature	N2	–
	Rainfall (A3)	Annual precipitation	–	Nature	N3	–
**Economy (B)**	Insurance (B1)	Unemployment insurance coverage	+	Economy	E2	–
	Financial expenditure (B2)	Financial expenditure per capita	+	Economy, Society	E3	–
	Economic diversity (B3)	Proportion of primary sector	–	Economy	E4	–
**Society (C)**	Education (C1)	University students per 10,000 population	+	Society	S1	s1
	Population (C2)	Population density	–	Society	S2	–
	Age (C3)	Minors and the elderly as a percentage of the total population	–	Society	S3	s2
	Basic medical care (C4)	Number of hospital beds per 10,000 people	+	Society	S4	s3
	Gender (C5)	Percentage of females	–	Society	S5	–
**Infrastructure (D)**	Roads (D1)	Density of roads	+	Infrastructure, Nature	I1	i2
	Water infrastructure (D2)	Investment in water infrastructure	+	Infrastructure, Economy	I2	i3
	Green area (D3)	Greening rate of built-up areas	+	Infrastructure	I3	i4
	Pipe networks (D4)	Density of pipe networks in built-up areas	+	Infrastructure, Nature	I4	–
	Communication (D5)	Mobile phone penetration rate	+	Infrastructure, Society	I5	i5

### 3.2 Determination of weighting coefficients

In this study, the weights of ten positive indicators and six negative indicators are established using the entropy method. The process for determining these weights involves the following steps: First, an indicator evaluation matrix is created using Formula (1). The target matrix is then normalized. Next, the entropy value of each evaluation indicator is calculated using Formula (2). Finally, the entropy value obtained from Formula (2) is inserted into Formula (3) to calculate the weight of the *j*-th indicator.

(1) $$X={\left\{x_{ij}^{t}\right\}}_{mT\times n}$$

(2) $$e_{j}=-\ln\frac{1}{mT}\sum_{t=1}^{T}\sum_{i=1}^{m}p_{ij}^{t}\;\ln\;p_{ij}^{t}$$

(3) $$W_{j}=\frac{1-e_{j}}{\sum_{j=1}^{n}1-e_{j}}$$

where $x_{ij}^{t}$ denotes the value of the *j*-th indicator for the *i*-th subject of evaluation in the *t*-th years, m represents the number of subjects evaluated, T symbolizes the number of years, n signifies the number of evaluation indicators, and $p_{ij}^{t}$ is the ratio of the *i*-th subject on the *j*-th evaluation indicator.

### 3.3 Calculation of the urban flood resilience index

To compute the urban flood resilience index, the weights derived in Section 2.2 are applied to (4), (5).

(4) $$r=\sum_{j=1}^{n}W_{j}y_{ij}^{t}$$

(5) $$R=r_{A}W_{A}+r_{B}W_{B}+r_{C}W_{C}+r_{D}W_{D}$$

where r denotes the resilience index of the indicator, $W_{j}$ is the indicator weight, R represents the urban flood resilience index, while $r_{A}$, $r_{B}$, $r_{C}$, and $r_{D}$ are the resilience values of the four aspects of natural, economy, society, and infrastructure, respectively.

### 3.4 Methodology of scenario simulation

For scenario construction, this study employs the sensitivity analysis method. By adjusting the growth rates of several key resilience indicators, we construct the required research scenarios. The altered values of these key resilience indicators are calculated using the specified formula.

(6) $$F^{\prime }\left(I\right)=F\left(I\right)+\sum_{t=1}^{n}F\left(I\right)•X_{\text{Ct}}^{I}$$

where $F^{\prime }\left(I\right)$ denotes the values of resilience indicators following fluctuations in growth rates, F(I) signifies the values of resilience indicators obtained from model predictions, and $X_{\text{Ct}}^{I}$ is the growth rate of indicator *I* for city *C* in *t*-th years.

When calculating the annual growth of key indicators, n=1, Formula (7) can be derived:

(7) $$F^{\prime }\left(I\right)=F\left(I\right)+F\left(I\right)•X_{C1}^{I}$$

in the context of predicting future data, the autoregressive moving average (ARIMA) model was used in this study to extract the statistical characteristics of future urban flood resilience indicator series, and the forecasting results were applied to the scenario simulation module of this study. The ARIMA model, as a tool that can generate effective synthetic series, has found extensive applications in predicting various data types, including population, urban green spaces, and flood occurrences [48,49].

### 3.5 Data sources

For our analysis, essential data pertaining to natural, economy, society, and infrastructural aspects were sourced from the Statistical Yearbook of Zhejiang Province and the statistical yearbooks of various cities within the province. Additional data on water infrastructure were obtained from the Zhejiang Provincial Department of Water Resources, and elevation data of the province were sourced from the Zhejiang Provincial Department of Natural Resources.

## 4 Resilience evaluation results

**Fig. 2:**
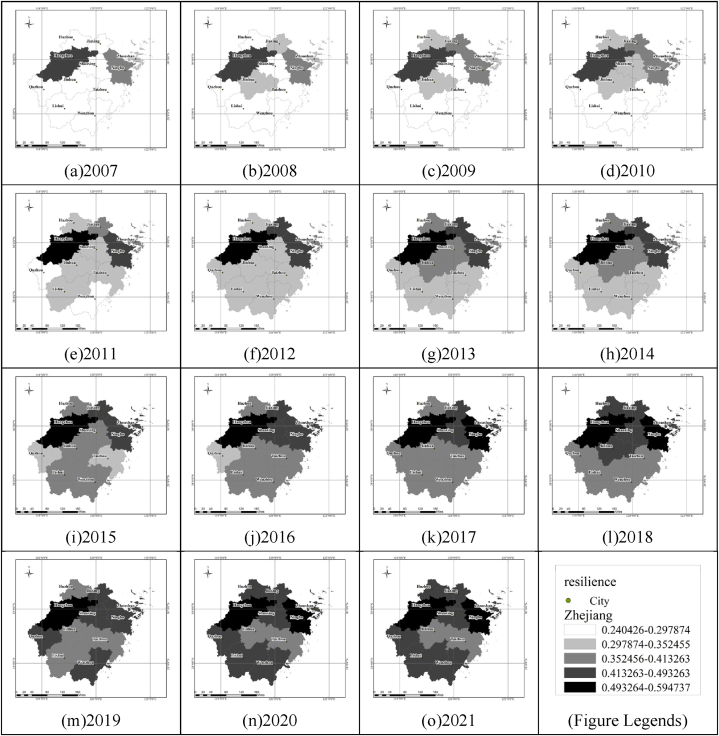
Urban flood resilience in cities of Zhejiang Province, 2007–2021.

The urban flood resilience of Zhejiang Province over the 15-year period from 2007 to 2021 was categorized into five levels using the natural breakpoint classification method, as depicted in Fig. 2. The analysis reveals a geographic pattern where urban flood resilience is higher in the northeast compared to the southwest of the province. Moreover, a progressive improvement in the flood resilience of each city over time was observed.

### 4.1 Analysis of the temporal dimension of resilience evaluation results

From a temporal perspective, Zhejiang Province's flood resilience has exhibited a 35 % increase from 2007 to 2021. Specifically, the annual average growth rate of flood resilience from 2007 to 2018 was 3.4 %. However, the COVID-19 pandemic in 2019 had a significant impact on the province's economic, infrastructural, and social development, resulting in a 9.2 % decrease in flood resilience compared to 2018. The period from 2019 to 2021 saw a gradual recovery, with an annual average resilience growth rate of 1.6 %. In addition, with the change of time, the resilience gap between cities in Zhejiang Province has been decreasing, and the resilience harmony has been increasing [50].

(1) Natural resilience. The natural environment significantly influences urban flood resilience. In Zhejiang Province, natural resilience has declined by about 20 % over the last 15 years, with 2021 marking the lowest level during this period. This decline is primarily due to land use changes, notably the expansion of urban construction land. By 2021, urban land expanded to 3955.7 km^2^, reducing the area's capacity to retain and detain floodwaters, thereby diminishing natural resilience.
(2) Economic resilience. Economic resilience in Zhejiang Province has notably improved, showing an average annual growth rate of over 20 % in the past 15 years. This enhancement is attributed to vigorous economic growth and the ongoing adjustment of industrial structures, leading to increased investment in areas like unemployment insurance and fiscal spending. The primary industry, which is particularly vulnerable to flooding, has been steadily decreasing at an average annual rate of 5.3 %. In 2021, it accounted for only 2.95 % of the economy. These shifts in the economic structure have significantly improved the region's ability to withstand flood disasters and facilitate recovery from them.
(3) Social resilience. Between 2007 and 2021, Zhejiang Province's social resilience remained relatively stable, with an average annual growth rate of less than 1 %. This stability is linked to the province's advanced educational and healthcare systems. Despite the annual 1.7 % increase in population density, which negatively impacted social resilience, improvements in education and healthcare have continuously enhanced the residents' disaster awareness, response, and recovery capabilities. In 2019, Zhejiang Province experienced the highest influx of population in the country, with approximately 840,000 individuals moving into the province. Furthermore, the COVID-19 pandemic had a significant impact on population movements. Restrictions on population outflow led to a higher growth rate in the permanent population density in 2019 compared to 2018, with an increase of approximately 10 %. At the same time, there was a temporary decline in the number of university students per 10,000 population, which had an impact on the residents' awareness and ability to respond to floods.
(4) Infrastructure resilience. The infrastructure resilience of Zhejiang Province demonstrated an average annual growth rate of 15 % between 2007 and 2021. This substantial growth can be mainly attributed to the province's dedicated efforts in developing municipal infrastructure and water infrastructure. Over this period, Zhejiang Province has consistently invested in enhancing road density, pipeline density, mobile phone penetration rate, urban green spaces, and water infrastructure. The progress in road construction and communication systems has bolstered the region's ability to effectively respond to and recover from disasters. Additionally, increased investments in pipelines, green spaces, and water infrastructure have not only lowered the likelihood of disasters but have also contributed significantly to the overall improvement of infrastructure resilience in the province.

### 4.2 Analysis of the spatial dimension of resilience evaluation results

Spatial analysis reveals distinct resilience patterns across Zhejiang Province. The five southwestern cities generally exhibit lower resilience compared to the six northeastern cities. Notably, Hangzhou consistently ranks as the most flood-resilient city in the province from 2007 to 2021. Conversely, Zhoushan, despite its relatively modest economic development, is among the top five resilient cities, attributed to its low population density, high per capita fiscal expenditure, and advanced education and healthcare systems.

(1) Natural resilience. The natural resilience of cities in the northeast region of Zhejiang Province surpasses that in the southwest region. This discrepancy can be primarily attributed to the relatively uniform distribution of precipitation throughout Zhejiang Province. However, there are substantial differences in terrain, with a noticeable tilt from the southwest to the northeast. The southwest region is characterized by mountainous terrain, leading to a higher flood risk, whereas the southeast region is predominantly flat, resulting in a lower flood risk. Among these cities, Jiaxing stands out as the city with the highest natural resilience in Zhejiang Province. In 2010, Jiaxing's natural resilience was 315.3 % higher than that of Lishui, the city with the lowest natural resilience. This distinction can be attributed to Jiaxing's predominantly flat terrain, which receives relatively less rainfall. The gradual and prolonged rainfall accumulation process in Jiaxing leads to the formation of fewer streams and makes it less prone to flood generation. In contrast, Lishui is primarily characterized by hilly terrain with steeper slopes and receives more abundant precipitation, resulting in a higher local flood risk. Consequently, Lishui ranks at the lower end of the natural resilience list.
(2) Economic resilience. The economic resilience is higher in coastal and riverside cities in Zhejiang Province compared to inland mountainous areas. Among them, Hangzhou and Ningbo stand out as the two cities with the highest economic resilience in Zhejiang Province. In 2021, Hangzhou and Ningbo had economic resilience scores of 0.157 and 0.145, respectively, securing the top two positions in the province. This is attributed to the relatively large proportions of the secondary and tertiary industries in these two cities, as well as a significant number of insured individuals in the insurance sector. Additionally, Hangzhou and Ningbo have higher per capita fiscal expenditures compared to other areas, which provides residents with increased resistance to flood disasters and quicker recovery in the aftermath of such events. Moreover, Lishui and Quzhou, characterized by smaller permanent populations and higher per capita fiscal expenditures, also enjoy relatively high rankings within the province. However, their economic resilience is relatively lower due to the underdevelopment of the secondary and tertiary industries and a lower number of insured individuals in the local region.
(3) Social resilience. The variation in social resilience across Zhejiang Province is relatively modest, with only about a 0.1 difference between the cities with the highest and lowest resilience over the past 15 years. This narrow range is largely attributable to the province's uniformly high levels of education and healthcare. Hangzhou, for instance, emerges as the city with the highest social resilience in the province. Factors contributing to this include Hangzhou's superior healthcare and education facilities, with the highest number of hospital beds per 10,000 people reaching 76, along with its relatively low population density and smaller proportion of vulnerable groups. These elements collectively strengthen the residents' awareness and adaptability in the face of flood disasters. Conversely, Lishui, while being the most resilient among the five southwestern cities, owes its resilience to lower population density and higher per capita healthcare and education levels.
(4) Infrastructure resilience. The disparity in infrastructure resilience among Zhejiang Province's cities is more pronounced, with the greatest difference being 0.087. Among these cities, Hangzhou emerges as the city with the highest infrastructure resilience in Zhejiang Province. Hangzhou enjoys certain advantages in terms of transportation, pipeline networks, and water conservancy investments, enabling the city to possess robust disaster perception and emergency response capabilities. Thus, Hangzhou's ability to respond promptly to flood disasters is well-established. Ningbo secures the second position in infrastructure resilience within the province. However, there exists a considerable gap between Ningbo and Hangzhou, with a difference exceeding 0.03 (Ningbo's highest infrastructure resilience is 0.03). This disparity primarily arises from the fact that, although Ningbo has a higher level of development in transportation and communication, its pipeline network density, green space ratio, and annual investments in water conservancy facilities are relatively lower compared to Hangzhou and other areas. Consequently, Ningbo's infrastructure resilience is notably lower. Lishui ranks at the lowest in infrastructure resilience in Zhejiang Province, primarily due to the limited development of water conservancy facilities, water supply and drainage pipeline networks, as well as communication and transportation infrastructure within the region. These deficiencies hinder Lishui's capacity to effectively respond to disasters.

## 5 Scenario simulation results

### 5.1 Scenario construction

Using the gray correlation analysis method, we calculated the correlation between various indicators and urban flood resilience values. From natural, society, economy, and infrastructure perspectives, key resilience indicators were identified based on these correlations. Notably, insurance, age, and communication demonstrated high correlation coefficients with urban flood resilience, recorded at 0.810, 0.887, and 0.893, respectively. Consequently, these indicators were selected as key resilience indicators. Four resilience scenarios were then developed to project the future development of urban flood resilience in Zhejiang Province over the coming decade.

#### 5.1.1 Base scenario (BS)

In this scenario, it is assumed that the growth of natural, society, economy, and infrastructure factors is standardized based on the trends observed over the past 15 years.

#### 5.1.2 Insurance awareness enhancement scenario (IAES)

Insurance plays a pivotal role in mitigating the influence of natural disasters on individuals and society, substantially bolstering their resilience. The heightened frequency of natural disasters has raised people's awareness of the associated risks, resulting in a greater proportion of individuals purchasing insurance [51]. In this specific scenario, it is assumed that urban residents' awareness of insurance has experienced a notable increase due to a combination of factors, including economic development and government policy support. Taking into account the differing developmental statuses of various cities and the defining characteristics of this scenario [52], the growth rate of key indicators has been determined, as outlined in Table 4.

**Table 4:** Growth rate of the number of insured individuals in various cities of Zhejiang Province under the IAES.

Growth rates of key indicators	City
4 %	Jinhua, Zhoushan
5 %	Hangzhou, Wenzhou, Jiaxing, Shaoxing, Taizhou
6 %	Ningbo, Huzhou, Quzhou, Lishui

#### 5.1.3 Fertility encouragement scenario (FES)

*The Outline of the Fourteenth Five-Year Plan for the National Economic and Social Development of Zhejiang Province and the Visionary Goals for 2035* proposes to promote the optimization of the population structure, improve the whole range of eugenics services and promote the construction of a fertility-friendly society [53]. Assuming that Zhejiang Province has been actively implementing pro-natal policies, leading to an additional increase in population birth rates, while the death rate has remained unchanged. As a result, there has been a further increase in the proportion of minors and elderly individuals. The growth rate of key indicators in this context, as mentioned in Table 5, has been determined accordingly.

**Table 5:** Growth rate of the percentage of minors and the elderly in various cities of Zhejiang Province under the FES.

Growth rates of key indicators	City
1 %	Wenzhou, Jiaxing, Huzhou, Shaoxing, Jinhua, Quzhou, Taizhou, Lishui
2 %	Hangzhou, Ningbo, Zhoushan

#### 5.1.4 Communication expansion scenario (CES)

Given the anticipated continuous increase in mobile phone penetration rates in Zhejiang Province, this scenario explores the impact of expanded communication capabilities on flood resilience by assuming an additional increase in this indicator. The growth rates of key indicators for this scenario are calculated according to the methodology outlined above and are enumerated in Table 6.

**Table 6:** Growth rate of mobile phone penetration rate in various cities of Zhejiang Province under the CES.

Growth rates of key indicators	City
1 %	Lishui
2 %	Ningbo, Wenzhou
3 %	Jinhua, Zhoushan, Taizhou
4 %	Hangzhou, Jiaxing
5 %	Huzhou, Shaoxing, Quzhou

### 5.2 Analysis of the scenario simulation results

**Fig. 3:**
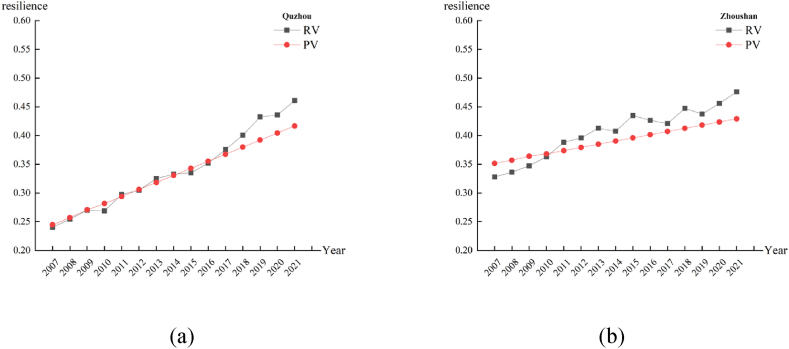
Comparison of projected and real values.

Comparing the predicted values with the actual values for the period 2007–2021 under the baseline scenario, most of the errors between the predicted values and the real values are within 5 %, which makes the prediction results reasonable. The predicted and real values for some cities are shown in Fig. 3.

In BS, Zhejiang Province has witnessed growth in natural, society, economy, and infrastructure resilience across various regions. Over ten years, both the overall flood resilience of the province (18.63 %) and the resilience of each city have significantly improved. Due to diverse levels of development across the regions, resilience disparities among cities have consistently exceeded 0.16. Hangzhou, with the highest resilience, is projected to reach a resilience value of 0.6131 b y 2031, 0.18 higher than Jinhua, the lowest in the same year. Notably, Taizhou, initially the lowest in resilience from 2022 to 2025, exhibits substantial growth in communication, per capita fiscal expenditure, and roads, surpassing Jinhua by 2026. This suggests that Jinhua could benefit from enhanced infrastructure development and increased fiscal investment to improve its flood resilience.

In IAES, the province's flood resilience has improved more compared to BS, with a ten-year growth rate of 18.67 %, slightly higher by 0.04 %. This improvement is linked to increased insurance awareness and coverage, enhancing post-disaster recovery capabilities. Quzhou displays the highest average annual growth rate in resilience (2.43 %) among the 11 cities. In this scenario, Taizhou exceeds Jinhua's resilience one year earlier than in BS.

In FES, the flood resilience in Zhejiang Province has demonstrated a continuous increase. This positive trend can be attributed to the implementation of the fertility encouragement policy, resulting in a 1 %–2 % increase in the proportion of minors and elderly individuals in various regions compared to BS. However, it is essential to note that the increased proportion of vulnerable groups who are threatened by flood disasters has led to a reduction in the overall resilience of cities to such disasters. Compared with other scenarios, the flood resilience extreme value in Zhejiang Province has slightly decreased in FES. It is 0.0022 lower than the highest extreme value observed in CES. This suggests that encouraging childbirth, while beneficial in some aspects, contributes to reducing the differences in flood resilience among different cities.

**Fig. 4:**
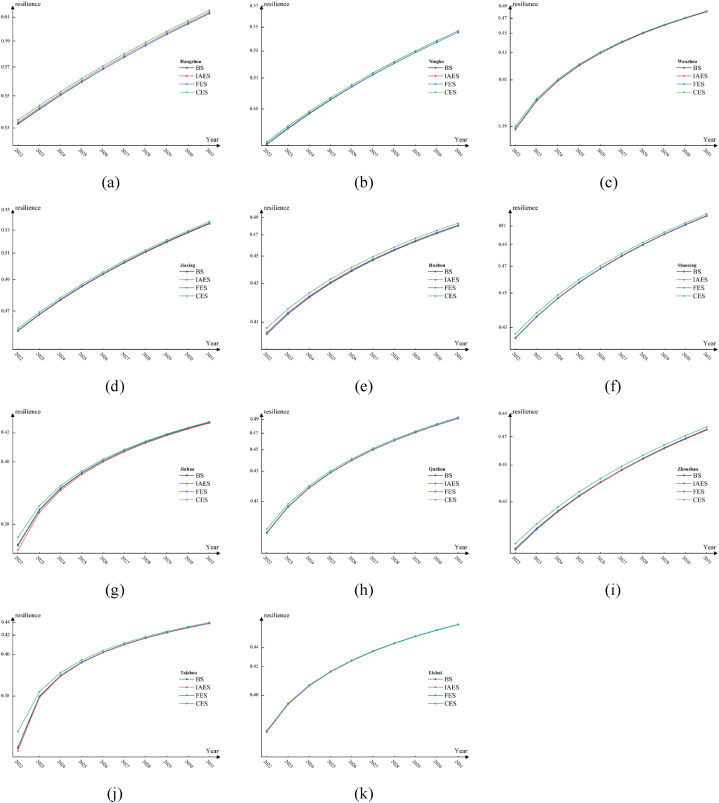
Comparison of flood resilience under different scenarios in different cities of Zhejiang Province.

In CES, both the overall flood resilience in Zhejiang Province and the flood resilience of each city within the province rank the highest among the four scenarios. This notable improvement can be primarily attributed to the substantial growth in mobile communication infrastructure in Zhejiang Province, which still has room for further development. Therefore, the increase in flood resilience is more evident in CES. For instance, in 2022, the flood resilience value of Hangzhou was 0.0022 higher than BS, 0.0014 higher than IAES, and 0.0025 higher than FES. Other cities also witnessed varying degrees of improvement in their resilience values. This positive trend can be attributed to the expansion and development of communication infrastructure, which effectively enhances residents' ability to receive timely information and reduces additional disaster losses induced by inadequate information dissemination [54]. The comparison of flood resilience results among different scenarios from 2022 to 2031 in various cities is depicted in Fig. 4.

## 6 Discussion

### 6.1 Indicator system construction and scenario setting

While flood disasters are bound to happen in certain cities, it is essential to determine the specific capacity of these cities to withstand floods by creating appropriate evaluation models. This is crucial for minimizing flood-related losses [7]. Nonetheless, when it comes to cities with varying characteristics, using existing methods to establish an evaluation system, such as summarizing and extracting information from prior studies [19,26], constructing it based on local attributes [13], or tailoring it to different stages of flood disasters [27], presents significant challenges. In this study, we have taken a comprehensive approach by considering the results of prior research, current national standards, and local characteristics to build a resilience evaluation model. The enhancement of the indicator system's scientific rigor, along with the refinement of its accuracy in resilience evaluations, offers a practical framework for constructing similar systems in other regions. This approach holds significant relevance for comparable research endeavors.

Zhejiang Province, placing a high premium on coordinated regional development, has yielded tangible outcomes. *The* 14th *Five-Year Plan for National Economic and Social Development in Zhejiang Province* emphasizes that the province has made some progress in achieving a higher level of coordinated regional development. This progress is reflected in the narrowing of the flood resilience gap across municipalities and is consistent with the results of this study. The resilience evaluation model we developed in this study identifies Hangzhou as the city with the highest flood resilience among those in Zhejiang Province, which aligns with previous research findings [55]. Additionally, the flood resilience of cities in Zhejiang Province exhibits an overall upward trend over time, no more low resilience level cities in Zhejiang Province after 2020 [56].

Previous studies on flood resilience have primarily focused on static evaluation [27], which may not fully correspond with the future development trajectories of affected areas. Introducing policy scenarios into resilience evaluation research can effectively test policy effectiveness and reduce disaster losses [57], providing a more dynamic and forward-looking approach.

### 6.2 Policy implication

(1) Cities like Hangzhou, with high flood resilience and dense populations, should prioritize infrastructure improvements. Enhancing drainage network standards, building underground pipelines, and renovating outdated drainage facilities can significantly reduce the vulnerability of urban systems to disasters [2]. Alternatively, by establishing decision support matrices for sensing systems applicable to different types of commercial buildings and utilizing sensors and other communication devices, the disaster sensing ability of residents can be improved while satisfying their living comfort [58].
(2) Emerging resilient cities such as Taizhou and Jinhua need to focus on increasing insurance awareness and communication coverage. Local governments should promote investment in insurance and communication through various channels, including propaganda, legislation, and financial incentives, issue a series of plans to improve urban resilience in disaster preparation and conduct regular inspections on their effects prior to disasters [59]. This strategy will improve residents' disaster awareness, response, and post-disaster recovery capabilities.
(3) In urban areas where a significant portion of the population is vulnerable, because vulnerable groups, such as older persons, are less receptive to systems that can improve disaster response, such as smart government services [60], it is essential for the government to take steps to improve the training and assistance offered to social organizations and vulnerable communities. This includes spreading information and teaching essential skills for effectively handling emergency situations [55], or through the construction of smart resident-centered government services [61]. Through the implementation of these initiatives, the government can empower local residents to better help themselves during crises and reduce the overall impact of disasters.
(4) To address the issue of declining birth rates and a shrinking population, Zhejiang Province should not only promote policies encouraging childbirth but also prioritize infrastructure development and financial investments. It is essential to enact supportive policies aimed at safeguarding the well-being and assets of vulnerable communities during flood incidents. This reduces geographic disparities in resilience and facilitates the realization of the policy of shared prosperity.

### 6.3 Limitations and future research

In this study, we evaluated urban flood resilience in Zhejiang Province and projected future development trajectories using scenario simulation. Unfortunately, data constraints precluded the inclusion of certain indicators related to social learning capacity, such as the frequency of emergency flood drills and historical disaster experience, in the development of the indicator system. In practice, social learning capacity does influence the flood resilience of a region. Moving forward, it would be beneficial to integrate these indicators into the indicator system to offer a more precise framework for guiding urban development and the construction of resilient cities.

## 7 Conclusion

This study introduces a more systematic approach to constructing an indicator system, develops a framework for evaluating urban flood resilience, and evaluates the dynamics of flood resilience in Zhejiang Province. Through scenario simulation, the study elucidates the development trends of flood resilience under various scenarios, enriches the methodology of resilience research. The principal findings are summarized below:

(1) The findings reveal notable disparities in flood resilience across Zhejiang Province, both in terms of time and space. In terms of time, over a 15-year period from 2007 to 2021, eleven cities in Zhejiang Province witnessed a substantial increase in flood resilience, exceeding 30 %. Notably, Quzhou exhibited the most remarkable surge in flood resilience, with a staggering growth rate of 69.5 %. Looking at the spatial aspect, there are significant variations in flood resilience among different regions within Zhejiang Province, with a pronounced degree of polarization. Hangzhou stands out as the city with the highest flood resilience in the province, while cities like Taizhou and others lag behind with a resilience value difference of over 0.2.
(2) In the scenario simulation, as time progresses, the flood resilience of Zhejiang Province continues to improve, experiencing an average annual growth rate of 1.86 %. Notably, the development of the communication and insurance industries plays a more prominent role in enhancing resilience values. Additionally, the implementation of childbirth encouragement policies, while increasing the proportion of vulnerable populations to some extent, has an impact on urban flood resilience development. However, it also serves to reduce the disparities in resilience between various cities, promoting more synergistic regional development.
(3) Regarding the methodology, the method of constructing the indicator system by taking into account prior scientific research, local attributes and applicable standards has improved the applicability and scientifice rigor of the urban flood resilience evaluation indicator system. The evaluation results obtained by constructing the indicator system through the above methods meet the actual needs of Zhejiang Province, enables a scientifically grounded evaluation of flood resilience development in the region and offers valuable insights for areas characterized by concentrated rainfall, lengthy coastlines, and frequent flood disasters. Furthermore, the incorporation of scenario simulations within the resilience evaluation enhances the study of dynamic urban resilience, broadening the scope of urban resilience research. This approach proves advantageous for long-term city planning efforts.
(4) Drawing from the evaluation of urban flood resilience and the results of scenario simulations, this study puts forward suggestions aimed at enhancing urban flood resilience. These recommendations are intended to assist local authorities in tailoring their efforts to the specific characteristics of their regions. Furthermore, they can use the insights from the scenario simulations conducted in this study as a reference point. By considering factors including natural, economy, society, and infrastructure, these recommendations enable focused flood control initiatives, reducing the adverse effects of floods on local areas.

## CRediT authorship contribution statement

**Feifeng Cao:** Project administration, Methodology, Funding acquisition, Conceptualization. **Hao Xu:** Data curation, Software, Visualization, Writing – original draft. **Guixia Huang:** Validation, Software. **Conglin Zhang:** Writing – review & editing, Supervision, Funding acquisition.

## Data availability statement

Data will be made available on request. For requesting data, please write to the corresponding author.

## Funding

This research was supported by the 10.13039/501100001809National Natural Science Foundation of China, grant number 12002310; the 10.13039/501100004731Zhejiang Provincial Natural Science Foundation of China, grant number LZJWZ23E090010; the Key Project of the 10.13039/501100004177Ministry of Water Resources of China: Research on the Guidelines and Measures for Flood Control Management in Territorial Space, grant number E2031019; and the Second Comprehensive Scientific Expedition to the Tibetan Plateau, grant number 2019QZKK0401.

## Declaration of competing interest

The authors declare the following financial interests/personal relationships which may be considered as potential competing interests:Feifeng Cao reports financial support was provided by the 10.13039/501100001809National Natural Science Foundation of China. Feifeng Cao reports financial support was provided by the 10.13039/501100004731Zhejiang Provincial Natural Science Foundation of China. Conglin Zhang reports financial support was provided by 10.13039/501100004177Ministry of Water Resources of the People's Republic of China. Conglin Zhang reports financial support was provided by 10.13039/501100002367Chinese Academy of Sciences. If there are other authors, they declare that they have no known competing financial interests or personal relationships that could have appeared to influence the work reported in this paper.
